# The process of overcoming conflicts among teachers in the implementation of comprehensive sexuality education at ordinary public senior high schools in Mataram City, Indonesia: a qualitative study

**DOI:** 10.1186/s41182-023-00495-y

**Published:** 2023-02-03

**Authors:** Fumiko Shibuya, Dian Puspita Sari, Cut Warnaini, Arina Windri Rivarti, Rie Takeuchi, Tracey Elizabeth Claire Jones-Konneh, Calvin de los Reyes, Hamsu Kadriyan, Jun Kobayashi

**Affiliations:** 1 Department of Global Health, Graduate School of Health Sciences, University of the Ryukyus, 207 Uehara, Nishihara-cho, Nakagami-gun, Okinawa, 903-0215 Japan; 2 Japanese Consortium for Global School Health Research, 207 Uehara, Nishihara-cho, Nakagami-gun, Okinawa, 903-0215 Japan; 3 Faculty of Medicine, University of Mataram, Jalan Pendidikan 37, Mataram, West Nusa Tenggara 83125 Indonesia; 4 College of Arts and Sciences, University of the Philippines Manila, 625 Pedro Gil Street, Ermita, Manila, Philippines

**Keywords:** Comprehensive sexuality education (CSE), Conflicts, Qualitative study, Indonesia

## Abstract

**Background:**

Comprehensive sexuality education (CSE), which aims to help young people make responsible choices and acquire scientific knowledge and skills, has been promoted by UNESCO. Teachers experience conflicts in implementing CSE when teaching sexual topics in the local context, especially as the delivery of sexual knowledge and contraceptive methods is often prohibited by religious and traditional cultural norms. It was reported that there were multiple challenges in the implementation of sex education due to the religious and cultural background of societies and communities in Islamic countries. This study aimed to clarify the process of overcoming the conflicts, explore teachers’ recognition and perception related to the implementation of CSE, and to suggest recommendations for promoting CSE in Islamic areas.

**Methods:**

This qualitative study combined the methods of focus group discussions (FGDs) and in-depth interviews (IDIs) to explore the conflict among teachers. Ten ordinary public senior high schools in Mataram City, Indonesia, agreed to participate, and in total, 59 participants were involved in this study. FGDs were conducted with teachers (*n* = 49), and IDIs were focused on school principals (*n *= 10) in each school. The collected interview data were analyzed using a deductive thematic analysis and the findings triangulated for both the FGDs and IDIs.

**Results:**

Overall, the teachers experienced conflicts in relation to religion, cultural background, and gender inequality in implementing CSE. The present study revealed the mutual recognition among teachers and acceptance of diverse backgrounds in the implementation of CSE at ordinary public senior high schools in Mataram City. Despite teachers reporting multiple conflicts, they made efforts to overcome these conflicts through mutual recognition and provided comprehensive guidance. The present findings indicated that teachers adapted CSE to follow multiple religions and cultural backgrounds.

**Conclusions:**

The teachers accepted diverse backgrounds and provided CSE by collaborating with related educational subjects and external institutions to overcome conflicts. To provide more specialized education, it would be necessary to advocate a formal policy that might be accepted by diverse societies. Further research is necessary to apply the findings and recommendations for CSE implementation globally in the contexts of different countries.

## Background

Comprehensive sexuality education (CSE), which was promoted by the United Nations Education, Scientific and Cultural Organization (UNESCO) in 2009, aims to help young people make responsible choices, develop appropriate sexual behavior, and acquire appropriate scientific knowledge and skills based on life cycle and culture [1, 2]. CSE includes scientific information about human development and reproductive health, as well as information about contraception, childbirth, and sexually transmitted infections, including HIV. Therefore, the promotion of CSE should involve cooperation with the whole school community because it includes not only sex education but also human rights and gender equity. CSE can provide enhanced decision-making and health literacy skills that are applicable to adolescents throughout the course of their life and may facilitate the acquisition of gender equity and human rights based on the appropriate developmental stage [3, 4]. Sex education is focused on sexual topics, such as the prevention of HIV/AIDS, the development of sexual organs, and menstrual education.

CSE can prevent risky sexual behavior and reproductive health issues among adolescents through comprehensive education on HIV/AIDS and other sexual transmitted infections and the prevention of unwanted pregnancy. CSE has the advantage of potentially preventing child abuse, early pregnancy, and sexually transmitted infections and also supports the understanding of gender and the building of good relationships [1]. A previous study provided evidence of the potential beneficial effects of CSE on attitudes, knowledge, and behavior regarding sexual and reproductive health among adolescents [5]. Further, previous studies reported that adolescents may acquire well-being and human rights by learning CSE, and knowledge and understanding of human rights could influence the acceptance of diverse backgrounds [4, 6]. Similarly, school-based sex education helped to enhance grades and reduce the incidence of school dropout due to early marriage and pregnancy [7].

Sex education in Islamic areas is associated with challenges with respect to the lack of a specific strategy and community-based educational programs provided by national and regional governments (e.g., unmarried women may not accept information about contraceptive methods or may avoid accessing information on contraception due to religious beliefs) [8, 9]. A systematic review focused on Muslim women’s sexual and reproductive health indicated that religious beliefs, tradition, and culture contribute to difficulty in applying contraceptive methods and accessing information [10]. Moreover, both religion and culture can be associated with these difficulties in Islamic societies, where certain social behaviors are prohibited or are considered to be unacceptable, such as extramarital sexual relations [8]. With regard to Muslim adolescents, talking about sexual content is still taboo between parents and educators due to religious norms and passive attitudes, and they also felt uncomfortable discussing reproductive health-related issues [11, 12]. Hence, sex education is still a sensitive topic in Islamic societies, and an appropriate program and teaching method should be provided to teach sexual content based on religious beliefs.

The Indonesian government initiated a school-based HIV/AIDS program in 1997, but the program was not formally incorporated into the official curriculum [13]. Experts on sexual and reproductive health were invited to deliver school-based HIV/AIDS education [14]. A school-based learning program was mandated by the United Nations to deliver sex education, and the sex education program in Indonesia is also mandated under the national policy. Nonetheless, Utomo and McDonald [12] and Utomo et al. [14] and Susanto et al. [15] reported that like many other countries, Indonesia still did not have a national curriculum related to sex education and that formal school-based sex education programs were still commonly considered taboo or controversial. In contrast, some parties have supported and strongly urged the government to include sex education as a part of the school curriculum. The Indonesian National Child Protection Commission (Komisi Perlindungan Anak Indonesia: KPAI) is one of the parties that have recommended government implementation of sex education at schools [16]. The KPAI proposed the inclusion of sex education in the school curriculum in 1999, but it was reported that in 2017 the government still had not responded to the proposal [16], and at the time of writing, this is still the case. Thus, Indonesia still has no national program, and the implementation of sex education has depended on teachers’ experience and perceptions based on existing subjects.

A previous study on sex education in Indonesia showed that the prevailing conservative views limited some information about sexual and reproductive health (SRH), and it was generally assumed that teenagers do not need to learn about SRH until they are married [17]. In contrast, the study found that the provision of SRH information among adolescents was enhanced by acquiring knowledge of the religion and that learning to manage sexual attitudes in a healthy and responsible manner possibly protected human rights [17]. Therefore, the study recommended conducting SRH education or promotion models accompanied by a religious approach to promoting sex education in Indonesia.

Teachers have conflicts in the implementation of CSE with regard to teaching sexual topics in the local context, especially as the delivery of sexual knowledge and contraceptive methods is often prohibited by religious and traditional cultural norms [18]. Some teachers are afraid that CSE promotes sexual curiosity and behavior among students [19] and that through CSE, adolescents could exhibit more active sexual behavior. Hence, teachers have conflicts in relation to cultural and religious values, national policies, and personal perceptions based on their experience as educators when teaching CSE. de Haas and Hutterb [18, 19] indicated that the guidance of sex education is related not only to educational institutions but also to national policy and traditional values in the community.

Previous studies in Islamic countries reported multiple challenges in the implementation of sex education due to the religious and cultural background of the society and community [9–11]. The issues of sexual and reproductive health are related not only to stigma relative to sexual content but also to comprehensive topics such as religious norms or the social environment. In previous studies, the implementation of CSE was related to the local context, which was mainly limited to Christian areas [18, 19]. Islam, which accounts for approximately 25% of the world’s population [20], has also been shown to be associated with a negative understanding of sex education practices [21]. Therefore, conducting a similar study to identify teachers’ conflicts in Islamic countries and to find solutions to them is expected to provide important insights for the future global promotion of CSE.

This study conducted in Indonesia, which has the largest Muslim population in Asia and is home to a diverse mix of ethnicities and religions, will not only consider the Islamic background of the country but will also explore how the country is embracing diversity and promoting CSE. A literature review did not identify any studies that focused on the process of overcoming conflicts among teachers in the implementation of CSE. Therefore, this study aimed to clarify how teachers overcome the conflicts that they experience in the implementation of CSE in schools and to explore how teachers recognize CSE and perceive its implementation in schools.

## Theory of conflict

In this study, we applied the theory of conflict to better understand the process of conflicts among teachers and how these conflicts are overcome for the implementation of CSE. The meaning of conflict was defined as the arousal of two or more strong motives that cannot be solved together in psychology [22]. The theory of conflict was advocated by Lewin, and *approach–avoidance conflict* refers to a decision or behavior that is simultaneously associated with desirable and undesirable consequences [23]. Thus, according to the theory of conflict, we hypothesized that conflicts among teachers would impact both their approach to and avoidance of conflict.

## Methods

### Study design

This study used a qualitative method, which triangulated focus group discussions (FGDs) and in-depth interviews (IDIs). Tolley et al. mentioned in 2016 that researchers conduct Qualitative Research when they need to search for a phenomenon that is not quantitative [24]. Further, the procedure of triangulation was applied to ensure the validity of the study through multiple qualitative data and gained consensus among researchers [25]. Hence, this qualitative study combined the methods of FGDs and IDIs to explore the conflict experienced by teachers in implementing CSE at schools.

### Sampling procedure

This study targeted ordinary public senior high schools in Mataram City, Indonesia. This target was chosen because most students in Indonesia are educated in ordinary (secular) schools. In the education system, ordinary schools fall under the jurisdiction of the Ministry of Education and Culture, whereas Islamic schools fall under the jurisdiction of the Ministry of Religion. These schools run in parallel at each educational stage from elementary school to university [26]. Approximately 80% of students in Indonesia are educated in ordinary schools, and the remaining 20% are educated in Islamic schools [26].

First, invitations to participate in this study were sent to all ordinary public senior high schools in Mataram City as the initial step in the recruitment process. Ten of the 11 ordinary public senior high schools agreed to participate in this study. Before recruiting participants for the data collection, we estimated the required number of participants to be five teachers and one school principal from each school. From the list of potential teacher participants provided by the schools that received an invitation to participate, the research team contacted the teachers, and informed consent was obtained from each teacher who agreed to participate.

Second, we targeted both the school principals and teachers at each school. Five teachers who are in charge of relevant CSE subjects were included in FGDs, and we selected teacher participants who were in charge of subjects relevant to CSE based on the inclusion criteria. The teachers’ subjects included: physical education, religious education, biology, civic education, and guidance and counseling. The participants were selected regardless of age because we could not obtain information on the teachers’ age. Moreover, the research team decided to limit the number of participants to only five in each FGD session to ensure optimum discussion within the allocated time. Also, the IDIs were conducted with school principals. Participants were selected based on their work experience and their availability to participate in online data collection.

### Participant characteristics

In total, 49 teachers (female, *n* = 24; male, *n* = 25) from 10 ordinary public senior high schools in Mataram City participated in the FGDs (Table 1). Their mean age was 41.3 years (range 21–57 years) (two non-responses were excluded). Most participants reported that they were Muslim; two participants reported that they were Hindu. The mean teaching experience was 14 years (range 1–30 years [one non-response was excluded]).

**Table 1:** Characteristics of the teachers (*N* = 49)

	n
Sex	
Male	25
Female	24
Age, years	
20–29	9
30–39	11
40–49	14
≥ 50	13
Non-response	2
Religion	
Islam	47
Hinduism	2
Buddhism	0
Christian	0
Other	0
Teaching experience, years	
0–9	14
10–19	24
20–29	8
≥ 30	2
Non-response	1
Position	
Vice-principal	4
Year-head teacher	27
Homeroom teacher	18
Subject taught	
Physical education	9
Religious education	10
Biology	10
Moran and civic education	9
Guidance and counseling	9
Sociology	2

For the IDIs, 10 school principals (female, *n* = 3; male, *n* = 7) participated in this study (Table 2). Their mean age was 49.1 years (range 39–56 years). The mean teaching experience was 21.6 years (range 11–31 years [one non-response was excluded]). All principals were Muslim.

**Table 2:** Characteristics of the school principals (*N* = 10)

	n
Sex	
Male	7
Female	3
Age, years	
20–29	0
30–39	1
40–49	3
≥ 50	6
Religion	
Islam	10
Hinduism	0
Buddhism	0
Christian	0
Other	0
Teaching experience, years	
0–9	0
10–19	3
20–29	4
≥ 30	2
Non-response	0

### Online data collection

Data collection for this study was conducted through FGDs and IDIs using an online methodology. FGDs were conducted to elicit the process of overcoming conflicts through discussions among teachers, whereas. IDIs were conducted to reveal detailed management-level challenges experienced by school principals. Ten FGDs and 10 IDIs were conducted with all participating schools between June and September 2021.

The FGDs were conducted at 10 ordinary public senior high schools in Mataram City based on the online protocol and interview guide. The average duration of the FGDs was 1 h and 53 min (range, 1 h and 38 min to 2 h and 30 min). We targeted the school principals with the IDIs because we presumed that we could clarify how teachers overcome the conflicts that they experience in the implementation of CSE in schools. Furthermore, we hypothesized that the role of the school principals and how they engage with the conflicts in the implementation of CSE at school could be explored. Thus, the IDIs targeting the school principals were conducted using specific questions to clarify any obstacle or challenge at the management level. The mean interview time was 51 min (range, 37 min to 59 min).

### Process of transcription and translation

The FGDs and IDIs were conducted in Bahasa Indonesia, and the interviews were transcribed by referring to online data processing that was developed through pretesting [27]. The research team from the University of Mataram was responsible for participant recruitment, conducting the data collection (FGDs and IDIs), and translating the qualitative data from Bahasa Indonesia into English. After every interview, recorded audio data of the Zoom meeting were transcribed using Google Docs by the principal investigator. Translation of the transcripts was conducted by the research team from the University of Mataram using both the Google Docs transcription and the recorded audio data of the Zoom meeting. These two materials complemented each other in the translation process and ensured the validity of the transcription. Furthermore, the Google Docs transcript reduced the time needed to transcribe the recording, and the recording helped to identify and correct inaccuracies in the transcript.

### Data analysis

The data from the FGDs and IDIs were analyzed using thematic analysis for qualitative study methods. In the data analysis of this study, we applied a deductive thematic analysis, as proposed by Boyatzis in 1998 [28]. We considered the predicted outcome and created the interview questions by referring to both technical guidance on CSE [1] and the previous study relevant to focusing on teachers’ conflicts in implementing CSE [18]. de Haas and Hutterb reported on the outcomes of studies that focused on teachers’ conflicts and their professional identities in comprehensive school-based sexuality education [18, 19]. Thus, we analyzed the qualitative data deductively and clarified the analyzed data compared with the previous study to evaluate the novelty of the present study.

**Fig. 1:**
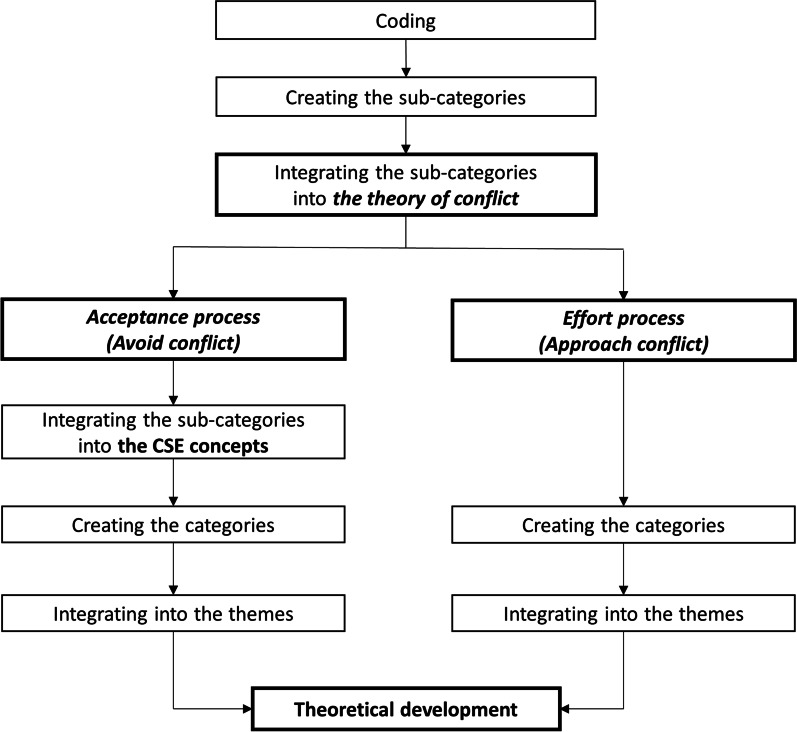
Flowchart of deductive thematic analysis

After data processing, data analysis was conducted by deductive thematic analysis methods. The data analysis process was divided into seven steps: coding, creating sub-categories, integrating sub-categories into the theory of conflict, integrating the sub-categories into CSE concepts, creating categories, integrating into themes, and theoretical development (Fig. 1). The coding was conducted using the MAXQDA 2018 Analytic Pro qualitative analysis software program [29]. Finally, the data analysis and theoretical development were regularly discussed with advisors and acquired a consensus that indicated agreement among co-researchers to reach reliability throughout each procedure.

### Trustworthiness of the study

Regarding the trustworthiness of the study, we tried to ensure reliability through online data collection and data analysis, and three classified procedures were mainly applied as follows. First, member checking/respondent validation: at the end of the FGDs and IDIs, we provided a summary of the discussion to the participants and asked them to confirm and correct our notes and interpretation. Member checking supports the credibility (internal validity) of a qualitative study. Second, triangulation: two sources of data were used in this study: teachers as the implementers and school principals who oversee and manage the teaching processes. The conflicts and challenges conveyed by teachers were confirmed by the principals. In addition, triangulation was also carried out in the data analysis. The members of both research teams, from the University of the Ryukyus and the University of Mataram, were involved in the data analysis. Triangulation of data sources and analysts supports the credibility and reliability of the study. Finally, to support the transferability of the findings, we already provided detailed information about the context of this study including the sample, setting, and results, so that the audience can evaluate how the findings are relevant to their contexts.

## Results

According to the theory of conflict, Lewin introduced three models of conflict “Approach–Approach conflict”, “Avoidance–Avoidance conflict”, and “Avoidance–Approach conflict” [23]. In this study, the conflicts among teachers in implementing CSE were adapted to the model of “Avoidance–Approach conflict” because the process of conflicts not only included the negative (Avoid conflict) but also the positive (Approach conflict). Thus, we applied the theoretical development to both conflicts such as “Avoid conflict—Acceptance process” and “Approach conflict—Effort process”.

A total of 155 codes were divided into 73 codes for “Acceptance process” and 82 codes for “Effort process”. Additionally, the percentages of the codes through two processes were 47.1% for “Acceptance process” and 52.9% for “Effort process”. This meant that “Acceptance process” and “Effort process” were approximately equivalent, and two processes of conflict were comparable among teachers. To integrate the sub-categories into the theory of conflict, the sub-categories were abstracted into this theory based on the two perspectives of “Acceptance process” and “Effort process”. Moreover, the sub-categories were divided into “Causes of the conflicts” and “Overcoming the conflicts” through theoretical development. Therefore, we analyzed the created themes through both the processes of acceptance and effort.

**Fig. 2:**
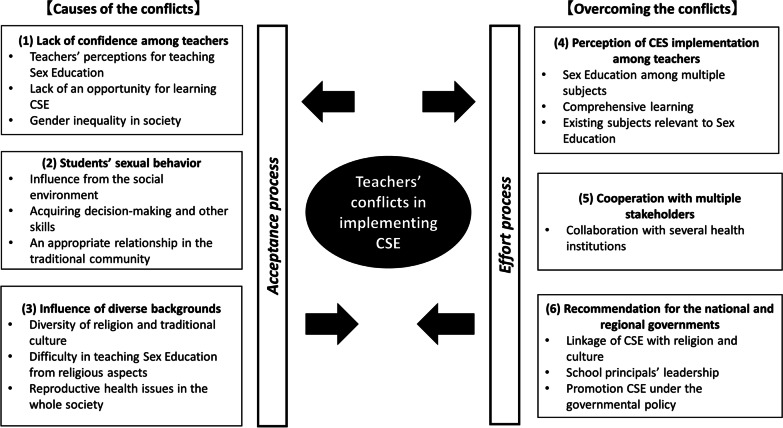
Concept mapping of these themes through two conflict processes

Six themes emerged from the FGDs and IDIs related to conflicts experienced by the teacher participants in teaching CSE and how these conflicts affected their teaching. These six themes were created from 16 categories and 36 sub-categories through Deductive Thematic Analysis (Table 3). Our analysis was based on two phases that focused on the process of teachers’ conflicts in implementing CSE, the “Acceptance process” phase and “Effort process” phase. “Causes of the conflicts” were represented by themes 1 to 3: (1) lack of confidence among the teachers, (2) students’ sexual behavior, and (3) influence of diverse backgrounds. “Overcoming the conflicts” were represented by themes 4 and 5: (4) perception of CSE implementation among teachers; and (5) cooperation with multiple stakeholders. Also, these themes explored the perceptions of teachers in implementing CSE in schools based on their experiences and the subjects they were in charge of. Furthermore, theme (6), recommendations for the national and regional governments, showed suggestions and opinions for promoting CSE (Fig. 2).

**Table 3:** Summary of themes and categories

Themes	Categories
(1) Lack of confidence among teachers	Teachers’ perceptions for teaching Sex Education
	Lack of an opportunity for learning CSE
	Gender inequality in society
(2) Students’ sexual behavior	Influence from the social environment
	Acquiring decision-making and other skills
	An appropriate relationship in the traditional community
(3) Influence of diverse backgrounds	Diversity of religion and traditional culture
	Difficulty in teaching Sex Education from religious aspects
	Reproductive health issues in the whole society
(4) Perception of CSE implementation among teachers	Sex Education among multiple subjects
	Comprehensive learning
	Existing subjects relevant to Sex Education
(5) Cooperation with multiple stakeholders	Collaboration with several health institutions
(6) Recommendation for the national and regional governments	Linkage of CSE with religion and culture
	School principals’ leadership
	Promoting CSE under the governmental policy

The created themes were developed based on the theory of conflict, which has two aspects of “*Acceptance process*” and “*Effort process*” as shown in Fig. 2. It refers to the theory of conflict that was advocated by Lewin in 1935.

### Lack of confidence among teachers

Teachers had no confidence in the implementation of CSE because their knowledge and perception about sex education were still incomplete. Most teachers had not experienced training in CSE under an official curriculum or guideline, and teaching sex education was dependent on the teacher’s approach:

“The teacher’s perception of sexual education is still biased. There are still pros and cons. But maybe the problem is because the teacher's understanding of sexual education is incomplete.” (School Principal)

“We face all sorts of difficulties at school. Some students are open to this topic, but some are quiet. It depends on how we approach them.” (Physical Education)

Some of the teachers mentioned a lack of opportunity to provide CSE under the curriculum or guidelines. In addition, formal CSE training has not yet been provided to educators:

“We don’t have a handbook for comprehensive sexual education. A handbook for anti-corruption education was included in the 2013 curriculum but there was nothing for comprehensive sexual education.” (Religious Education)

“I haven’t received formal training. I received training once when I was the supervisor for the student council. It was not a CSE-related training, but it was similar.” (School Principal)

The teachers indicated that a lack of gender equity was an obstacle in the provision of sexual topics from a gender perspective because they are not yet open about sexual-related matters due to gender inequality:

“Gender inequality still happens in Lombok. I give the students the understanding that boys and girls are the same.” (Sociology)

“I think maybe this is a sensitive topic that not all women feel open to discuss. Male students may be more open with male teachers. It is an obstacle for us to provide the best solution to sexual issues since we are not yet open about sexually related matters.” (School Principal)

### Students’ sexual behavior

Some of the teachers were afraid of encouraging sexual behavior among students outside of school due to the development of social media, which has influenced the understanding of sexual information among students. Further, students come from multiple backgrounds, and therefore, teachers must consider different religions and family situations when teaching sexual content:

“Students are now smarter than their teachers because they are very active on social media and have a wide range of interactions. So, when we talk about matters related to sexual education, the students already understand all about those things.” (Sociology)

“Students these days have a high degree of curiosity. They know more than us, even before we tell them. It's all because of modern technology. All students have a cell phone, so it's not difficult for them to obtain information.” (Sociology)

Some teachers reported being afraid that students learn sex education outside of school because it must be accomplished through moral education and norms. Moreover, some teachers mentioned that due to the delay in delivering sex education to high school students, their curiosity might encourage sexual behavior and misunderstanding. To avoid learning sex education outside of school, it seems that students should acquire decision-making skills to emphasize the importance of religion, norms, and morals:

“We hope that the students do not learn sex education outside of the school environment, which we are afraid of. Therefore, it should be emphasized that sex education in schools must be accompanied by moral education and norms.” (School Principal)

“Puberty starts at 13 years of age, it's safe to say that well-informed parents could give some input on that, but if the parents perceived this kind of talk as a taboo, they may be too embarrassed to talk about such issues, then it will be too late to deliver such information.” (Biology)

Many teachers noted that the traditional culture still supports early marriage in Lombok Island because students’ sexual behavior is related to traditional culture in the Sasak community, which still promotes early marriage. Some of the teachers felt it difficult to have a good relationship with students and family due to the multiple backgrounds:

“From the cultural perspective, in Lombok we have “merariq”. Merariq is snatching a bride. Some will consider this to be something that is easy or normal to do, but from a human rights perspective, even when women’s nature is to give birth, they also have the right to feel satisfied. That’s what we need to teach to our students, their rights and boundaries.” (Civic Education)

“Since there are many examples of underage marriage in our society, most students understood the consequences. Almost every child that married young has dropped out of school.” (School Principal)

The teachers mentioned that sex education was needed for adults too because if parents have appropriate knowledge, they can help their children. In addition, a bad relationship between students and parents impacted students’ attitudes and morals, and this underlying factor was associated with disharmonious relationships with parents:

“I usually emphasize the aspect of relationships, such as having a harmonious relationship with their family, to children because for students who have a lot of problems at school, the underlying factors are related to disharmonious family relationships. Bad relationships within the family at home will also affect the students at school…” (Guidance and Counseling)

### Influence of diverse backgrounds

This theme titled “influence of diverse background” related to how other religious and cultural backgrounds interacted with Islam or Sasak culture in influencing CSE or conflicts in teaching CSE. It was mentioned that the diverse cultural and religious background in Indonesia influenced the implementation of CSE. Some of the teachers taught about *Zina* in religious regulations through a lecture on religious education:

“We need to set the boundaries in interacting with people according to their respective religions. I believe that every religion prohibits Zina (unlawful sex).” (Religious Education)

“Contraceptive devices are one of the birth control methods. But in Islam, it’s forbidden to use such devices when you're not married.” (Religious Education)

Although the teachers are afraid of students possibly misunderstanding sexuality, some of them emphasized that morals and faith might be contributing to the enhancement of sexual topics that were still taboo, and sex education caused conflicts due to religious and cultural norms:

“What we must instill in our students, firstly, is faith and morality. If their morals and faith are good, they can avoid things that are not good. So, the first thing we instill is morals and faith.” (Civic Education)

“…people have often misunderstood this sexual education. This is as far as I understand as a teacher who has been in the midst of students for so many years. So, the fear arises in society because of their inadequate knowledge of sexuality, I think that's the root of the problem.” (School Principal)

Sexual content is still regarded as a sensitive topic in the local context, making it an obstacle to considering a solution to some sexual issues with the teacher. Talking about sex education openly caused uncomfortable feelings, and this discomfort hindered the teacher from discussing sexuality-related lessons:

“When talking about sex or human anatomy, we face the problem at school because some might feel embarrassed or uncomfortable talking about that topic so blatantly. Not everybody is used to talking about such a subject in such a vulgar manner.” (Guidance and Counseling)

### Perception of CSE implementation among teachers

Theme 4 indicated that CSE is implemented through multiple subjects based on the common perception among teachers because almost teachers acknowledged providing CSE to students to prevent their risky sexual behavior. According to the interviews with teachers, Sex Education is taught in Physical Education (HIV/AIDS, prevention of free sex/promiscuity) and Biology (sexual and reproductive health from a biological perspective). Furthermore, Religious Education (religious perspective about sex and relationships), Civic Education (Sociology), Moral Education (Pancasila and Character Education), and Guidance and Counseling were supported for students to gain an appropriate sexual attitude and life skills (Table 4):

“…we teach healthy relationships between teenagers. The material includes the prevention of early marriage. The material in 10th grade physical and health education is mainly about how we maintain social interaction it through physical education approaches, such as using sports as a diversion.” (Physical Education)

**Table 4:** Structure of CSE implementation based on eight concepts

No.	CSE concepts	Subjects	Educators	Teaching methods
1	Relationship	• Religious Education (Islam and Hindu) • Civic Education (Sociology) • Moral Education (Pancasila/Character Education) • Guidance and Counseling	Educators who are in charge of each subject	• Discussion of moral issues among students (e.g., gender inequality, traditional culture) • Building a relationship from a religious perspective
2	Values, Rights, Culture and Sexuality			
3	Understanding Gender			
4	Violence and Staying Safe			
5	Skills for Health and Well-being			
6	The Human Body and Development	• Physical Education • Biology	Educators who are in charge of each subject	• Discussion of health issues among students (e.g., HIV/AIDS, unwanted pregnancy) • Learning about physical development based on the Physical Education textbook • Contraceptive methods based on the Biology textbook
7	Sexuality and Sexual Behavior			
8	Sexual and Reproductive Health			

A biology teacher taught students about the use of contraception through a lecture to deliver some options for contraceptive methods. Another teacher recognized the importance of delivering sexual topics to students, which are expected to contribute to their future:

“There is a theory regarding contraception in Biology... Based on my understanding, I also explain that there is natural contraception, such as the calendar method. So, I do not encourage or discourage one particular contraceptive. I want the students to know that they have options. I think it is important to teach them about it so that they will know the options for contraception when they become mothers later.” (Biology)

Religious education included CSE content to avoid misunderstanding and trouble among students. A religious education teacher noted that CSE has the same purpose and concept as Islamic study:

“We explain what would happen if we commit adultery, including the two categories of adultery, the social punishment and the religious punishment, the impact on their families and the community. We also explain how to avoid promiscuity or adultery, for example, by participating in positive activities, such as extracurricular activities at school and religious activities and also by maintaining a safe relationship with friends.” (Religious Education)

Content on relationships with family and good attitudes toward religious norms were presented by civic education teachers. Indonesian society has rights and cultural values, which are acknowledged based on the principle of the Pancasila:

“There are rights and cultural values in our society. We live in a heterogeneous society with many different backgrounds and characters. I told them the importance of holding on to Pancasila (the five principles of the Indonesian State). The first principle, Belief in The Mighty God, is important because it covers the other four principles. When we understand our religion, we know that every action is followed by its consequences.” (Civic Education)

A guidance and counseling teacher emphasized that support for students is important for their learning process because the support system provided by teachers influenced students’ concentration and psychological stability. A public senior high school organized a religious counseling guidance team to support students who had problems:

“The sole purpose is for students to receive psychological support to stay focused so that their teaching and learning process remains normal… That is why we teach them about free sex or juvenile delinquency, its impacts, and how to deal with it.” (Guidance and Counseling)

“If there is a problem, usually the homeroom teacher gives advice first, then gradually the BK (Guidance and Counseling) teacher and later the religion teacher. At this school, I form a team called the religious counseling guidance team, so this is a collaboration between homeroom teachers, BK teachers, and religious teachers to provide understanding to students.” (School Principal)

A teacher noted that character building or character education helps students to understand the situations or the environments. They can adjust themselves accordingly, which allows students to solve problems by themselves when they occur:

“Regarding character building, we hope that they can understand their environments, including the school environment, their own home environment, and their social environment as students. In counseling, we collaborate a lot with subjects, such as Pancasila and Civics, religion, Indonesian language, as well as other fields.” (Guidance and Counseling)

Social relationships with family and good attitudes toward religious norms must be developed. One teacher mentioned that students should follow religious norms in daily life, and family interaction is quite essential for young people to have good relationships in the community and society:

“Every morning starts with people praying together according to their believe, Hindus in one class and Muslims in another class. We use that occasion to approach the students, not just about sex education but also about social relationships and so on.” (School Principal)

### Cooperation with multiple stakeholders

A health worker working in primary health care was invited to give socialization lessons on adolescent reproductive health. The primary health care doctor explained the human reproductive system and reproductive health, and teachers hoped that students would share information with each other:

“We invited from primary health care workers several times for socialization about adolescent reproductive health, but not all students attended. Usually, only representatives could attend, such as the student council, the class president, or the leaders of extracurricular activities.” (School Principal)

Some teachers explained about school cooperation with non-government organizations or agencies that focus on health education, particularly in relation to sex education and HIV/AIDS:

“PLAN International is an organization that promotes children’s rights and equality for girls. They helped us to give lectures to our students… They also held virtual meetings with the students. Their lectures are related to comprehensive sexual education. The teachers you have interviewed before also slip in materials related to comprehensive sexual education in their classes, especially Biology and Islamic studies. There are programs from the government to introduce it at school.” (School Principal)

### Recommendations for the national and regional governments

It was emphasized that piety, faith, and morals are essential in living life, and schools need to work together with parents and families to help students develop their understanding of sexuality issues. The traditional community is still promoted early marriage, such as the traditional *Sasak culture* of Lombok Island:

“In Lombok, the religion and culture are in line. The existence of CSE is helpful in the community, especially regarding “merarik kodeq” (early marriage). Hopefully, government policies are positively in line with comprehensive sexual education.” (Physical Education)

“The concept of introducing CSE should be disseminated and socialized more, so, that it is more easily accepted in society.” (Biology)

An intervention by the religious leader provides social values and mutual assistance in the community. The religious approach was recommended by the school’s principal because sex education is related to religious norms:

“We need to involve government officials, local youth organizations, and religious leaders related to morals and norms. They have big roles in educating the general public.” (School Principal)

“Religious leaders also deliver comprehensive sexuality-related materials and their related social values, such as mutual assistance. Mutual assistance or helping each other is still practiced by the community in order to achieve harmonious relationships within the community.” (School Principal)

A civic education teacher suggested implementing CSE under a policy such as an *Umbrella Act*, which refers to a set of regulations or legal instruments from the government. One school principal mentioned that educational methods should be improved through a curriculum or learning tool:

“…it is important to have an “Umbrella Act” (a set of regulations/legal instruments as the basis) for its implementation, because if there is no law about it, it would be difficult to be implement.” (Civic Education)

“I think we need to have a sustainable plan, so it is also necessary to involve the government in providing socialization of comprehensive sex education as continuous learning.” (Religious Education)

A guidance and counseling teacher mentioned the necessity of a CSE handbook because there was no trusted information source regarding CSE. The development of an official guideline may be an informative information source:

“For example, if we relate it to the regulation of the Minister of Education and Culture, which prohibits bullying and violence, perhaps sexual education can be included in it or become the legal basis for implementing this CSE. So, according to the Ministry’s rules, there are already guidelines; it’s just a matter of how the school uses it to make programs like what we’re talking about right now.” (School Principal)

“I think that a handbook is what we need. This handbook will become a guide for the teachers…by having this handbook, we don’t have to search the internet anymore. It will also become easier to deliver it to the students because we have a trusted source.” (Guidance and Counseling)

School principals need human resources to help implement CSE because educators must acquire appropriate knowledge and teaching skills. Implementation of teacher training or workshops was recommended when the development of guidelines was discussed with a school principal:

“Regarding the implementation of this CSE, for human resources, teachers in the school should be given some kind of workshop or training related to this CSE concept so that they can collaboratively develop guidelines later on.” (School Principal)

## Discussion

The purpose of this study was to clarify how teachers overcome the conflicts that they experience in the implementation of CSE in schools and to explore how teachers recognize CSE and perceive its implementation in schools. The study revealed mutual recognition among teachers and the acceptance of diverse backgrounds in the implementation of CSE at ordinary public senior high schools in Mataram City, Indonesia. According to the results, these two findings identified that the sub-categories synthesized the findings in the theory of conflict based on two perspectives “*Acceptance process*” and “*Effort process*” throughout the theoretical development.

First, we identified that boundaries need to be set in interacting with people according to their respective religions through the *acceptance process* because each religion has diverse norms and practices to follow among the public in the region. Second, this study identified the mutual recognition among the teachers that they implemented CSE based on their respective fields, roles, and expertise. Because most teachers, as educators, acknowledged that early marriage and unwanted pregnancy should be avoided among students, the perception among teachers was that CSE could presumably prevent these reproductive health issues, and this challenge encouraged their motivation to implement CSE. Furthermore, it was acknowledged that multiple subjects were related to the implementation of CSE and that teachers cooperated with the school community, society, and multiple stakeholders. Therefore, the two main findings indicate that teachers adapted CSE to follow multiple religions and cultural backgrounds.

Diverse backgrounds, students’ sexual behavior, and lack of confidence among teachers were causes of conflict in implementing CSE. Multiple religions and ethnicities co-exist in Indonesia, and the government advocates *unity in diversity* [30]. Many of the teachers were afraid to teach sex education because sex is still a taboo topic, and religious norms and traditional culture still strongly exist in the community. It should be noted that *unity in diversity* is associated with teachers’ perceptions. In Mataram City, religious norms are related to both Islam and Hinduism [31]. Moreover, the traditional Sasak culture still promotes early marriage among young people in the community [32, 33]. Some of the teachers mentioned that the traditional practice influenced students’ active sexual behavior and was associated with dropping out of school. They made efforts to solve students’ health issues, given their different backgrounds and perceptions. From these findings, it was indicated that the teachers’ backgrounds were based on the country context.

This study showed that moral education is comprehensively provided through several subjects, including civic education based on Pancasila (the state ideology or principles of the state’s philosophy in Indonesia) [34], religious education, and guidance and counseling. Moral education was recommended by the Indonesian government to enhance students’ morals and the protection of their rights, and it was advocated based on the Long-Term Development Plan for 2005–2025 [35]. Furthermore, religious education could increase awareness of religious beliefs and practices and influence the personal, family, and community spheres [36, 37] because religious beliefs are related to both perspectives of sexual and reproductive health and religious norms. Therefore, moral education might enhance students' attitudes through the comprehensive curriculum that was integrated into civic education (Pancasila) and religious education.

Several health institutions are already collaborating to guide sex education in ordinary public senior high schools, such as the Health Department, primary health care workers, international NGOs, and the National Family Planning Coordination Board. PLAN International promoted CSE under sexual and reproductive health and rights, and it might promote the acceptance of CSE in society [38]. A private health sector intervention relevant to sexual and reproductive health and rights was implemented in Europe [39]. The CSE curriculum and guideline in the Netherlands were developed by Rutgers International, which is an organization in the private sector that implements activities based on the national policy and statements from several agencies [40]. In addition, CSE training was provided to educators by Rutgers International based on the created curriculum and guidelines. It was previously reported that the private sector provides many sexual and reproductive health activities in low-middle income countries [41]. The CSE policy paper reported by UNESCO suggested that cooperation between both the health sector and private sector could link school health services [42]. Further, private sectors may cover areas of the education sector lacking the intervention. According to the international technical guidance on sexuality education, it was developed to support an organization in both education and health for promoting sexuality education programs in and out of school. One recommendation would be to involve several parties, not only an organization in both education and health, but also one that cooperates with private sectors at the national and school community level. Hence, it was suggested that linkage with the private sector should be pursued to promote CSE in the community and society.

This study indicated that promoting CSE may require cooperation with the community and society, leadership from school principals, and the implementation of CSE under a formal policy. First, it would be recommended to involve both the traditional community and parents due to the influence of traditional practices. Second, through the leadership of school principals, the acceptance of CSE could be promoted by encouraging cooperation among schools, the community, and society, as a previous study indicated that school principals play a critical role in providing school direction and determining the culture based on the context for each school [43]. Moreover, the leadership of school principals could contribute to the realization of health-promoting schools and help foster cooperation with the school community and stakeholders [44]. Finally, it was indicated that a policy would be needed to formally promote socialization of CSE. It was considered that CSE was implemented by mutual recognition among teachers, but to provide more specialized education, it would be necessary to advocate a formal policy that might be accepted by diverse societies. Thus, this study showed that the national and regional government would need to advocate a policy relevant to CSE.

As one limitation of this study, further research needs to be carried out to apply the findings to other countries. Although this study was undertaken to explore the global promotion of CSE, it was strongly related to the country context at the study site. While the original Indonesian educational system and diverse backgrounds are associated with the main findings of this study, the findings may not apply to the context of another country because CSE is related to religious norms, cultural backgrounds, and gender perspectives, and adapting all of the findings to another setting would be difficult. In addition, this study suggested that the difficulties of CSE need to be weighted for each challenge or suggestion because it was necessary to clarify the levels of issues (school, community, or national or regional government) related to CSE. In the process of the data analysis, themes were generated at the school, community, and national and regional government levels, but the priority of recommendations was not clear. Hence, it is recommended that further research be conducted in other locations based on the country and local context, and it will be necessary to make recommendations according to the priorities of each location.

## Conclusion

The conflicts experienced by teachers in Mataram City, Indonesia, in the implementation of CSE were mainly related to religion, cultural background, and gender inequality. Despite teachers reporting multiple conflicts, they made efforts to overcome these conflicts through mutual recognition and provided comprehensive guidance. Thus, the teachers accepted the diverse backgrounds of their students and provided CSE by collaborating with related educational subjects and external institutions to overcome conflicts. Further research is necessary to apply the findings and recommendations for CSE implementation globally in the contexts of different countries.

## Data Availability

Not applicable.

## Abbreviations

CSE: Comprehensive sexuality education
FGDs: Focus group discussions
IDIs: In-depth interviews
KPAI: Komisi Perlindungan Anak Indonesia
SRH: Sexual and reproductive health
UNESCO: United Nations Education, Scientific and Cultural Organization
